# ALK+ Anaplastic Large Cell Lymphoma With Bladder Involvement Presenting as Fever of Unknown Origin: A Case Report and Literature Review

**DOI:** 10.4021/wjon644w

**Published:** 2013-05-06

**Authors:** Joshua Lawrenz, Justin Tomal, James Towne, Beth Johnson, Brent Rieger

**Affiliations:** a Division of General Internal Medicine, Department of Medicine, Loyola University Medical Center, 2160 S. First Ave. Maywood, IL 60153, USA; b Department of Pathology, Central DuPage Hospital of Cadence Health, 25 N. Winfield Rd. Winfield, IL 60190, USA

**Keywords:** Anaplastic large cell lymphoma, Bladder, Fever of unknown origin, Macrophage activation syndrome

## Abstract

Anaplastic large cell lymphoma (ALCL) is a rare malignant tumor normally originating in lymph nodes, though it can occur in extranodal sites. We report a 59-year-old man with anaplastic lymphoma kinase (ALK) positive ALCL involving the bladder diagnosed post-mortem who presented with fever of unknown origin. This is the seventh reported case of ALCL presenting as a bladder neoplasm. The patient presented to his primary care physician with a several day history of fever. An eventual computed tomography scan of the abdomen and pelvis showed widespread adenopathy in the pelvis and retroperitoneum. After a negative infectious investigation, the patient underwent exploratory laparotomy with excisional biopsy of periaortic lymph nodes. Pathology revealed reactive lymphocytes. Bone marrow biopsy also was negative for malignancy. The patient’s fevers persisted, and he later exhibited dysuria and hematuria with evidence of bilateral hydronephrosis. Cystoscopy revealed an erythematous, diffusely friable bladder mucosa with inaccessible ureteral orifices, and biopsies were taken. The patient continued to deteriorate clinically because of associated macrophage activation syndrome, a close variant of hemophagocytic lymphohistiocytosis, and expired the following day. Autopsy was declined. Post-mortem pathology reports from cystoscopy revealed ALK+ ALCL of the bladder.

## Introduction

Fever of unknown origin (FUO) is a condition that has been associated with considerable challenges in work-up and diagnosis. It was first defined by Petersdorf and Beeson in 1961 with the following criteria: fever higher than 38.3 °C on several occasions, duration of fever for at least three weeks, and uncertain diagnosis despite one week of appropriate investigation in the hospital [1]. The differential diagnosis of FUO classically has been divided into three major subgroups: infections, connective tissue diseases and malignancies. The most frequent occult malignancies to cause FUO are of reticuloendothelial origin, including leukemia and lymphoma. Frequently, lymphoma is diagnosed with bone marrow biopsy. Furthermore, nodal site involvement can be identified with physical examination or advanced imaging, such as computed tomography (CT) scan or magnetic resonance imaging (MRI), followed by diagnostic biopsy.

We report the case of a 59-year-old man presenting with FUO that signified a challenge in establishing an underlying etiology. This case illustrates how an extensive work-up of FUO, including bone marrow biopsy and excisional nodal biopsy do not necessarily rule out the diagnosis of lymphoma in cases of extranodal origin. Although our patient’s final diagnosis of anaplastic lymphoma kinase (ALK) positive anaplastic large cell lymphoma (ALCL) with bladder involvement is rare, it is important to maintain malignancy high on the differential in a FUO presentation as many cancers have a favorable prognosis with proper treatment.

## Case Report

**Figure 1:**
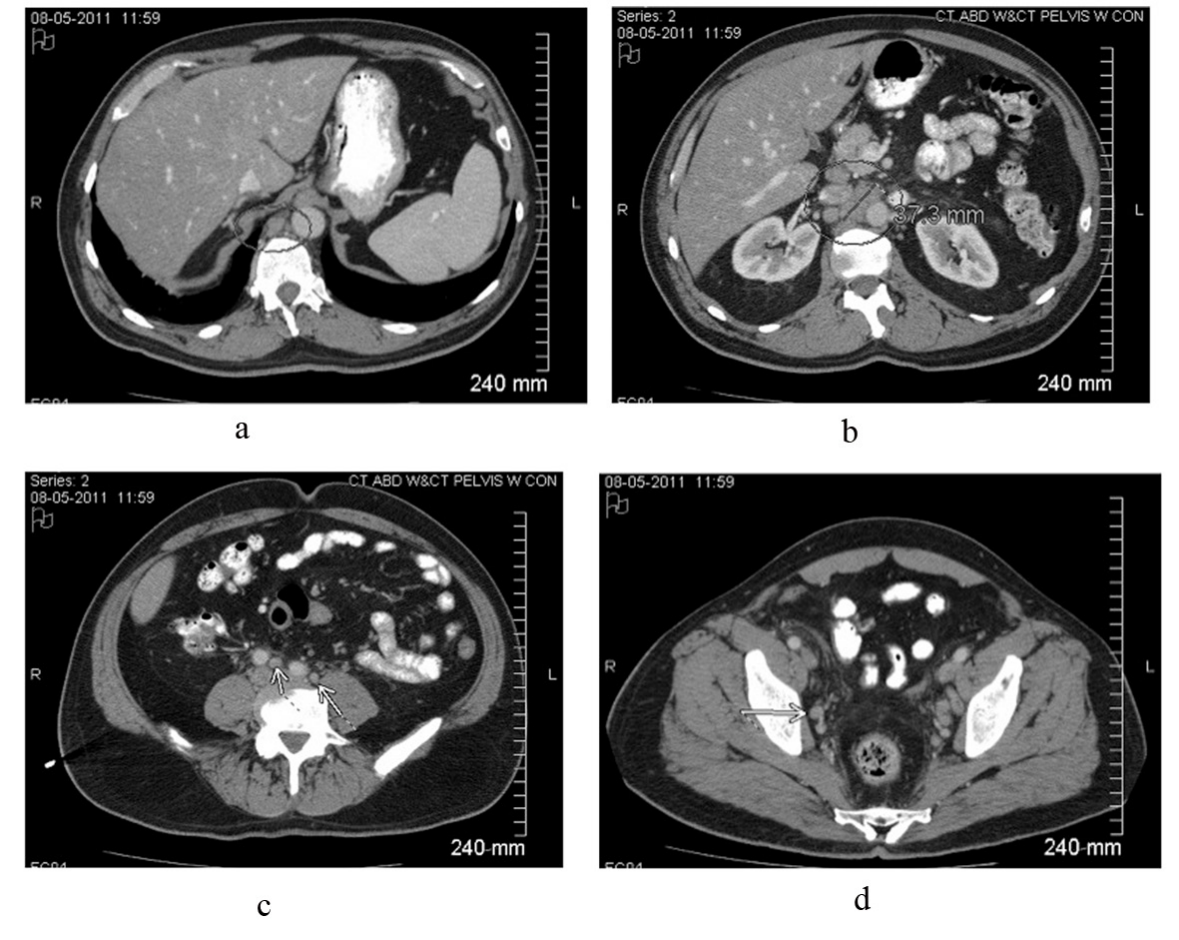
Contrast-enhanced CT scan of the abdomen and pelvis reveals intrabdominal and retroperitoneal adenopathy of uncertain origin. Adenopathy includes right retrocrural, retroperitoneal, celiac, peripancreatic, mesenteric, iliac and pelvic lymph nodes. Upper retroperitoneum reveals confluent nodal mass measuring 37.3 millimeters in image b.

A 59-year-old white male presented to his primary care physician with 4 days of fever (about 38.3 °C), chills, mild dry cough and mild right flank discomfort without dysuria. His past medical history included gastroesophageal reflux disease and nonalcoholic steatohepatitis, and there was no family history of malignancy. Urinalysis demonstrated small leukocyte esterase and trace blood. The patient was started on a course of ciprofloxacin. Urine culture was ultimately negative, and ciprofloxacin was discontinued. Chest roentogram raised suspicion for left lower lobe infiltrate, and he was placed on azithromycin; however, daily fevers of 38.8 - 39.4 °C persisted. A CT scan of the abdomen and pelvis revealed widespread adenopathy including a confluent retroperitoneal nodal mass measuring 4 cm (Fig. 1). Biopsy was deferred pending further work-up for presumed infectious etiology.

**Figure 2:**
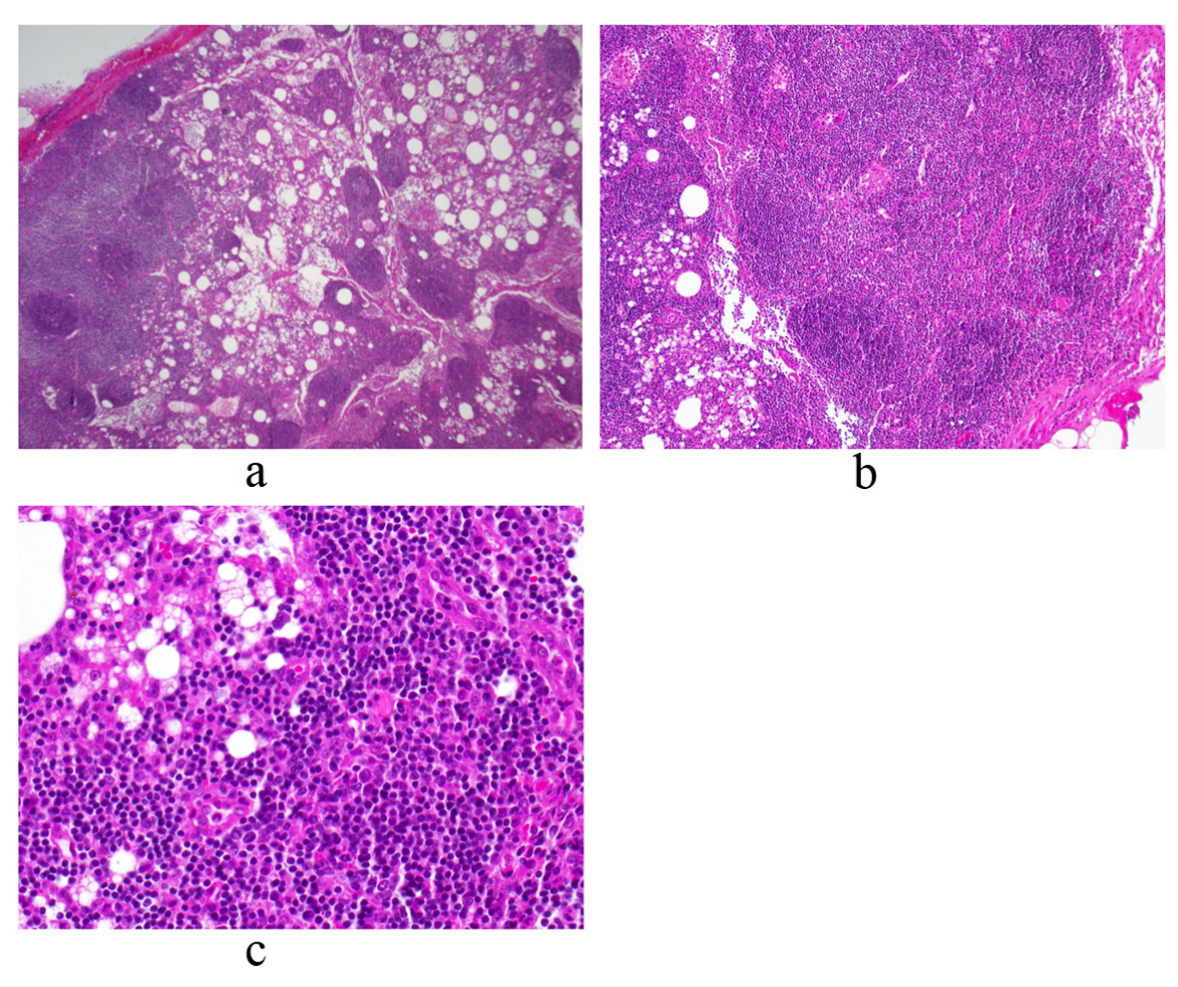
Periaortic lymph node biopsy reveals a mature and polymorphous lymphoid population with some macrophages and normal histologic architecture. There is an intact capsule and no lymphocyte migration outside of the capsule. There are follicles with germinal centers and normal interfollicular cellularity. Photos a, b and c represent low, medium and high power views, respectively.

On day 11, the patient was admitted to a community hospital for continued fatigue, malaise, and fever as high as 39.6 °C. Pertinent initial labs included leukocytosis (18.6 × 10^9^/L, 80% neutrophils), hemoglobin within normal limits (Hb = 13.6), elevated erythrocyte sedimentation rate (ESR = 65), elevated C-reactive protein (CRP = 12.9), elevated transaminases (AST = 62, ALT = 65), trace protein on urinalysis, negative blood and urine cultures, and negative monospot. Initial working diagnosis was FUO, including a differential of infectious, malignant and inflammatory etiologies. Further laboratory and imaging diagnostic modalities were completed (Table 1, 2, respectively). The patient continued to spike daily fevers, as high as 39.4 °C. Labs remained notable for leukocytosis (about 15 - 18 k) and mild transaminitis (AST/ALT in 100 s). On day 14, the patient underwent exploratory laparotomy with excisional biopsy of the 4 cm periaortic nodal mass found on previous CT. Light microscopy revealed normal histology with reactive lymphocytes (Fig. 2). Flow cytometry of a portion of the lymph node was also performed and found to be negative for any clonal B-cell or atypical T-lymphoid populations. On day 18, bone marrow biopsy showed hypocellularity, and the aspirate revealed granulocyte hyperplasia but no cellular atypia. Flow cytometry was negative for any atypical lymphocytes. The patient defervesced for the next thirty hours, but his temperature spiked to 39.4 °C on day 20. He continued to have fevers without a diagnosis, and was transferred to a tertiary hospital for further evaluation.

**Table 1:** Diagnostic Laboratory Work-Up Performed at Initial Hospital Admission

	Lab	Result		Lab	Result
Inflammatory	ESR	65 (H)	Infectious	PPD	Neg
	CRP	12.9 (H)		ASO	Neg
	C3	132		Hepatitis panel	Neg
	C4	30		Legionella urinary antigen	Neg
	RF	< 20		Parvovirus IgG	Wnl
	LDH	315 (H)		Parvovirus IgM	
Antibodies	ANA	+ 1:160		Coccidiodes antibody	Neg
	dsDNA	Wnl		CMV IgG	Wnl
	SS-a/SS-b	Neg		CMV IgM	Wnl
	Scl-70	Neg		CMV PCR	Undetectable
	RNP	Neg		EBV IgG	5.05 (H)
	Sm	Neg		EBV IgM	0.01
	Sm/RNP	Neg		EBV PCR	Undetectable
	C-ANCA/P-ANCA	< 1:20		Lyme Disease PCR	Undetectable
Endocrine	TSH	1.42		Malarial smear	Neg
	Free T4	1.8		HIV screen	Neg
	A.M.-Cortisol	27		Histoplasmosis urinary antigen	Neg
	SPEP	No monoclonal protein		Blastomycosis urinary antigen	Neg
	AFP	1.7		Blood cultures × 6	No growth
	B-HCG	1.7		Urine cultures × 2	No growth

Wnl: within normal limits; Neg: negative. ESR: erythrocyte sedimentation rate; CRP: C-reactive protein; C3/C4: complement; RF: rheumatoid factor; LDH: lactate dehydrogenase; ANA: anti-nuclear antigen; dsDNA: double stranded DNA; SS-a/b: Sjogren’s syndrome; Scl: scleroderma; RNP: ribonucleoprotein; Sm: Smith; C/P-ANCA: cytoplasmic/perinuclear antineutrophil cytoplasmic antibodies; TSH: thyroid stimulating hormone; T4: thyroid hormone; SPEP: serum protein electrophoresis; AFP: alpha-fetoprotein; B-HCG: beta-human chorionic gonadotropin; PPD: purified protein derivative; ASO: anti-streptolysin titer; Ig: immunoglobulin; PCR: polymerase chain reaction; EBV: Ebstein-barr virus; HIV: human immunodeficiency virus.

**Table 2:** Diagnostic Imaging Performed at Initial Hospital Admission

Image	Result
CT chest w/IV contrast	Neg. for mass or lymphadenopathy. Lungs clear.
Renal Ultrasound	Bilateral complex renal cysts: 2.9 cm in right kidney and 1.8 cm in the left kidney.
Transthoracic Echocardiogram	- Normal cardiac chamber size. Normal LVSF, EF 60%. Mild concentric LVH. - Trivial MR. Mild TR, estimated RVSP of 42 mmHg suggesting mild pulmonary HTN. - Small circumferential pericardial effusion w/o evidence of tamponade.
Nuclear Medicine Bone Scan	Nonspecific uptake in the right superolateral bony orbit.
Bilateral Lower Extremity Venous Doppler	Negative for deep venous thrombosis.

Neg: negative; LVSF: left ventricular systolic function; EF: ejection fraction; LVH: left ventricular hypertrophy; MR: mitral regurgitation; TR: tricuspid regurgitation; RVSP: right ventricular systolic pressure; HTN: hypertension.

At the time of transfer, the patient’s complaints were fever, chills, increased urinary frequency, mild post-operative abdominal pain and constipation. Pertinent labs included leukocytosis (12.5 × 10^9^/L, 90% neutrophils), anemia (Hb = 10.5) elevated ESR (40), elevated CRP (11.7), elevated transaminases (AST = 155, ALT = 89), elevated alkaline phosphatase (208), elevated lactate dehydrogenase (313), elevated total and direct bilirubin (2.5 and 1.3, respectively) and hypoalbuminemia (1.9). Urinalysis indicated 1+ protein, moderate blood (42 RBCs) and trace leukocyte esterase (WBC 10/HPF). Repeat cultures were negative.

Given the unrevealing work-up for infection and malignancy, rheumatology was asked to evaluate for an inflammatory or connective tissue disorder that could explain the patient’s continued symptoms. Based on high ferritin levels (2,213), mild arthralgias, continued fever, lymphadenopathy, and mild transaminitis, rheumatology suspected Adult-onset Still’s disease (AOSD). Although not meeting strict Yamaguchi criteria for AOSD, the patient was started on prednisone 1 mg/kg daily, and he was discharged home on day 27.

Despite prednisone, the patient continued to have fevers, and so he was readmitted to the hospital on day 30 with dehydration. The ferritin level was now 8,924, up from 2,213. The patient received IV solumedrol 50 mg twice/daily, and he was started on anakinra 100 mg subcutaneous daily, an interleukin-1 inhibitor. The patient continued to complain of lower urinary tract symptoms with blood clots now present in the urine, prompting Foley catheter placement. After an episode of gross hematuria the following day, CT of the abdomen and pelvis was ordered, demonstrating bilateral hydronephrosis with mesenteric, periportal and retroperitoneal lymphadenopathy. On day 35, cystoscopy was scheduled to place bilateral ureteral stents to relieve obstruction, but this was postponed due to new-onset atrial fibrillation.

**Figure 3:**
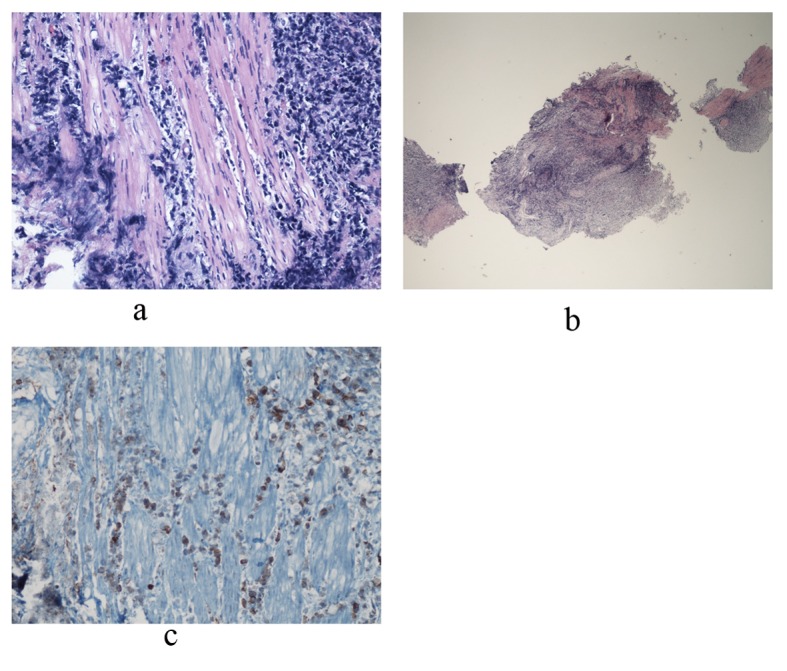
Bladder biopsy sections reveal a dense infiltrate composed of large abnormal lymphoid cells at low and medium power views. Photo a shows diffuse, ragged infiltration of bladder muscle by cells with dark nuclei and moderate size. An example of these cells is represented in photo b. Immunohistochemical stains showed that these large cells are positive for CD45, CD3, CD5, CD4, and CD30. CD30 positive cells are represented in photo c. Occasional large cells are also positive for ALK-1. They are negative for CD20, CD8, CD34, Tdt, CD56, pankeratin, synaptophysin and chromogranin. Findings are consistent with an ALK+ anaplastic large cell lymphoma.

The patient’s overall condition began to worsen rapidly, requiring intubation and pressors. Given the rapid clinical decline, the treatment team had concern for possible macrophage activation syndrome (MAS), evidenced by fever, hepatosplenomegaly, anemia, thrombocytopenia and hyperferritinemia with levels rapidly climbing to 49,713. MAS was managed with tocilizumab, cyclosporine, and intravenous steroids. On day 38, urology attempted to place ureteral stents to relieve bilateral hydronephrosis. Cystoscopy revealed an erythematous, diffusely friable bladder mucosa. The urologist was unable to visualize ureteral orifices, but random bladder biopsies were taken. Over the next 24 hours, hypotension persisted despite maximal support. At the family’s request, code status was changed to “do not resuscitate,” and pressors were withdrawn. On day 39 of his illness the patient expired, and an autopsy was declined. Several days later, the final pathology report of the bladder biopsy revealed ALK+ ALCL (Fig. 3).

## Discussion

ALCL is a subtype of the relatively rare peripheral T-cell lymphomas (PTCL), which comprise about 12% of non-Hodgkin’s lymphoma (NHL) [2]. Based on data from the International T Cell Lymphoma Project, which analyzed 1314 cases of PTCL or natural killer T-cell lymphoma (NKTCL), 12.1% were of the ALCL subtype [3]. ALCL may be subdivided into primary cutaneous ALCL and primary systemic ALCL. An important distinction of primary systemic ALCL is whether the cancer is ALK positive or negative as these entities have different clinicopathologic characteristics, with ALK+ status typically occurring in younger patients and conferring a more favorable prognosis [4]. ALK expression is detectable in about 60% of cases [5]. Regardless, both subtypes of ALCL usually have a characteristic morphology of large cells with horseshoe-shaped nuclei and almost universal expression of CD30 [3]. The most common translocation resulting in active ALK is t(2;5), whereby the fusion of ALK on chromosome 2p23 and the nucleophosmin (NPM) gene on chromosome 5q35 leads to a constitutively active tyrosine kinase that results in antiapoptotic behavior, although numerous other translocations have been described [5].

The majority of ALK+ ALCL patients present with painless lymphadenopathy and B symptoms (fever, night sweats, weight loss) as an aggressive stage III or IV disease [6]. One study showed extranodal involvement in 60% of cases, with the most common sites being skin (21%), bone (17%), soft tissues (17%), bone marrow (11%), lung (11%), and liver (8%) [7]. Primary lymphoma presenting in the bladder is a rare, though well-documented phenomenon, but the majority of cases are of the mucosa-associated lymphoid tissue (MALT) type [8]. A PubMed review of the literature yielded only 6 reports of ALCL presenting as a bladder neoplasm [9-14]. All 6 of these cases involved men between the ages of 22 - 45 years old. All but one presented with genitourinary symptoms, such as hematuria, scrotal swelling, or renal failure. One patient presented with severe lumbar pain [10]. While one HIV+ patient expired 9 months after diagnosis, the other 5 patients were alive and well at time of their respective papers’ publication after multiple cycles of CHOP (cyclophosphamide, doxorubicin, vincristine, prednisone), highlighting the favorable prognosis of ALCL with bladder involvement if diagnosed and treated promptly.

Our patient displayed only minor urinary tract symptoms (early right flank pain and later increased urinary frequency) throughout most of his course. It wasn't until the fifth week of his course when he had an episode of gross hematuria, which at the time was attributed to a traumatic foley placement from the day prior. Especially in light of multiple negative urine cultures, these symptoms were overshadowed by a FUO presentation that progressed to possible MAS, a closely similar syndrome to hemophagocytic lymphohistiocytosis (HLH). Indeed, FUO can delay an underlying malignant diagnosis through an extensive infectious and rheumatologic workup. One study at Emory University found 23 cases of ALCL from 1999-2006 at its institution, 5 of which did not have a correct pre-mortem diagnosis [15]. The majority of these patients presented with FUO, leukocytosis and thrombocytopenia. Additionally, 3 of these 5 patients had lactic acidosis, a condition not described in prior cases of ALCL, and 2 of 5 patients had HLH, which has been reported rarely with ALCL. Our patient had all of these characteristics and likewise was not diagnosed pre-mortem. As this study concludes, evidently a subset of patients with ALCL present with a nonspecific FUO and HLH-like picture that quickly progresses and complicates accurate diagnosis.

### Conclusion

ALK+ ALCL rarely may present as a bladder neoplasm and/or with FUO and HLH. Especially in clinical scenarios of the latter without an underlying diagnosis, leukemia and lymphoma must remain high on the differential diagnosis. In light of the rapidly aggressive course of ALCL presenting with HLH, and ALCL’s favorable response to CHOP, it is important to diagnose the underlying cancer early and initiate treatment promptly.
